# Supplementary material for: YprA family helicases provide the missing link between diverse prokaryotic immune systems
Source: bioRxiv. 2025 Sep 15:2025.09.15.676423. Preprint. [Version 1] doi: 10.1101/2025.09.15.676423 (PMC12458937; doi:10.1101/2025.09.15.676423)
Supplement: Supplement 4 [file NIHPP2025.09.15.676423v1-supplement-4.pdf]

bioRxiv preprint doi: https://doi.org/10.1101/2025.09.15.676423; this version posted September 15, 2025. The copyright holder for this preprint (which was not certified by peer review) is the author/funder, who has granted bioRxiv a license to display the preprint in perpetuity. It is made available under aCC-BY 4.0 International license.

Table of Contents

Page	Figure title
1	**Supplementary Figure S1: Comparison of Dpd with BRIGADE systems found in halobacteria**
2	**Supplementary Figure S2: Experimental and AF3 predicted structures of MrfA and DrmAB**
3	**Supplementary Figure S3: Complete efficiency of plating (EOP) results for the ARMADA anti-phage protection experiments shown in Figure 2**

bioRxiv preprint doi: https://doi.org/10.1101/2025.09.15.676423; this version posted September 15, 2025. The copyright holder for this preprint (which was not certified by peer review) is the author/funder, who has granted bioRxiv a license to display the preprint in perpetuity. It is made available under aCC-BY 4.0 International license.

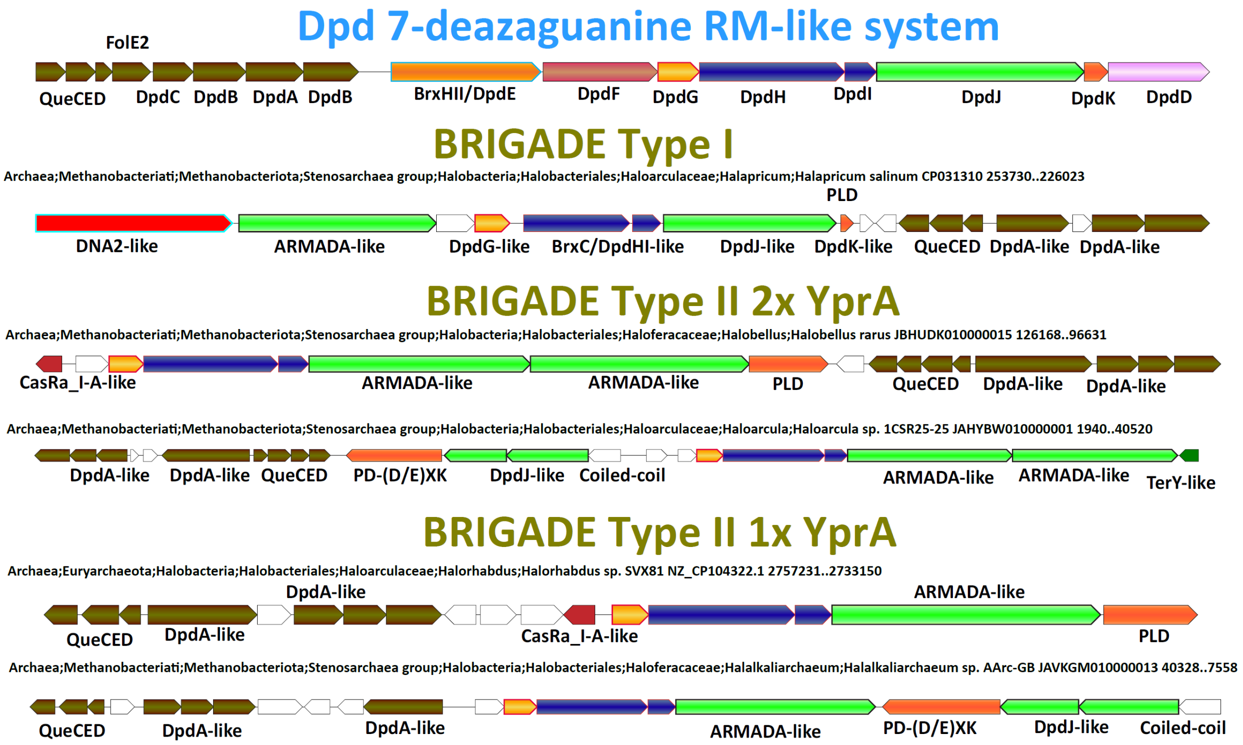

Supplementary Figure S1. Comparison of Dpd with BRIGADE systems found in halobacteria

Canonical Dpd systems encompass a 7-deazaguanine derivative biosynthesis and insertion operon, as well as BREX-like factors DpdE/BrxHII and DpdHI/BrxC (BrxHII is shared with DISARM and ARMADA), a YprA family helicase (DpdJ), and additional factors involved in restriction of non-hypermodified DNA. BRIGADE systems share many of these factors, including many, but not all of the genes in the 7-deazaguanine derivative biosynthetic cluster, DpdG, DpdHI/BrxC, at least one YprA homolog, and PLD superfamily endonucleases. BRIGADE Type II also usually includes a CasRa-like regulatory protein and has an alternative nuclease in an operon with a DpdJ-like YprA homolog when the PLD nuclease is not present. In contrast, BRIGADE Type I is almost always associated with a small PLD nuclease and often with a large DNA2-like helicase/nuclease fusion protein.

bioRxiv preprint doi: https://doi.org/10.1101/2025.09.15.676423; this version posted September 15, 2025. The copyright holder for this preprint (which was not certified by peer review) is the author/funder, who has granted bioRxiv a license to display the preprint in perpetuity. It is made available under aCC-BY 4.0 International license.

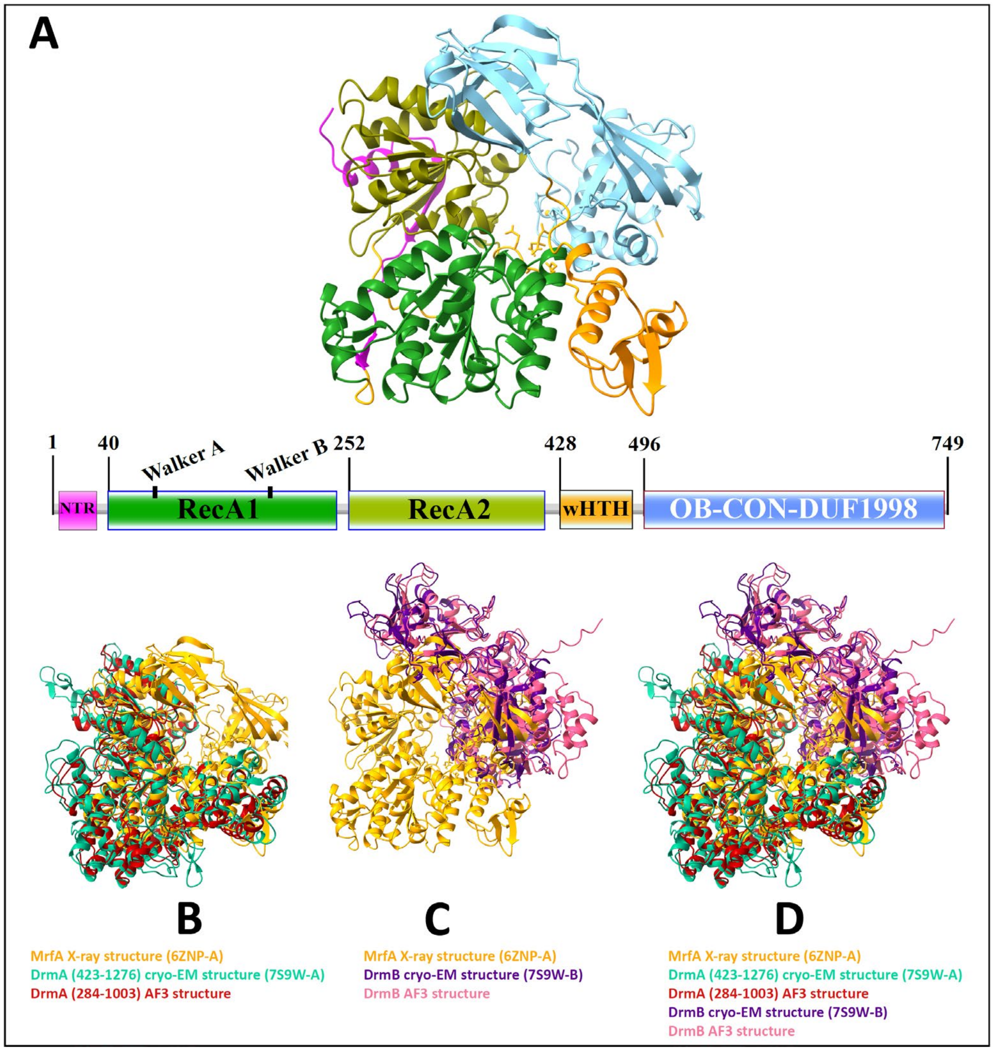

Supplementary Figure S2, Experimental and AF3 predicted structures of MrfA and DrmAB

(A) MrfA X-ray crystallographic structure and representation of domain architecture. The domains annotated are the N-terminal region (NTR, magenta), the first RecA-like domain containing the Walker A/B motifs (RecA1, dark green), the second RecA-like domain (RecA2, olive), a wHTH domain (orange), and the canopy-forming OB-CON-DUF1998 domain array (blue). (B) Superpositions of MrfA (gold), the DrmA helicase structure solved in complex with DrmB (teal), DrmA only shown here with N and C-terminal truncations for clarity, and a representative DrmA AF3 predicted structure (red), with similar N and C-terminal truncations. (C) Superpositions of MrfA (gold), the DrmB OB-CON-DUF1998 structure solved in complex with DrmA by Bravo et al. (purple), DrmB only shown here for clarity, and a representative DrmB AF3 predicted structure (pink). (D) Superpositions of MrfA (gold), the DrmAB helicase structure solved by Bravo et al. (DrmA: teal, DrmB: purple), with N and C-terminal truncations of DrmA for clarity, a representative DrmA AF3 predicted structure (red), with similar N and C-terminal truncations, and a representative DrmB AF3 predicted structure (pink).

bioRxiv preprint doi: https://doi.org/10.1101/2025.09.15.676423; this version posted September 15, 2025. The copyright holder for this preprint (which was not certified by peer review) is the author/funder, who has granted bioRxiv a license to display the preprint in perpetuity. It is made available under aCC-BY 4.0 International license.

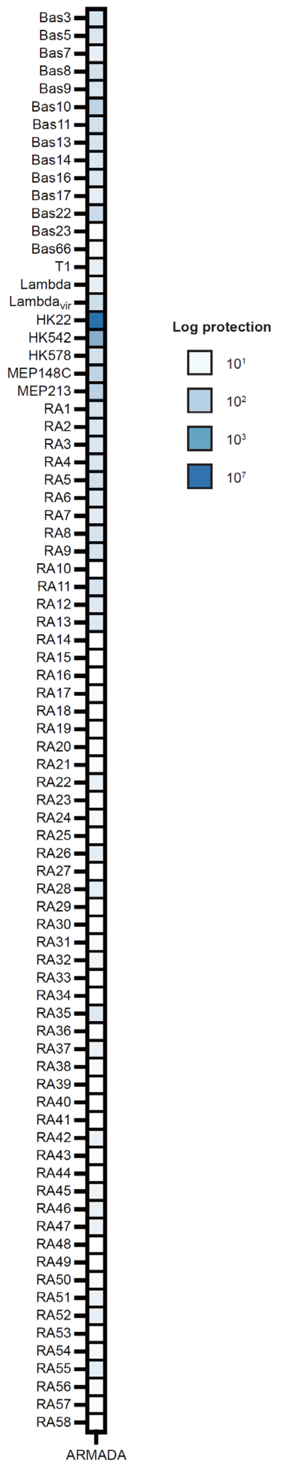

Supplementary Figure S3. Complete efficiency of plating (EOP) results for the ARMADA anti-phage protection experiments shown in Figure 2

Log-protection (reduction in phage titer) conferred by ARMADA, expressed from plasmid pBeloBAC11 in BL21-AI, against a panel of 80 phages
